# The role of the arousal system in age‐related differences in cortical functional network architecture

**DOI:** 10.1002/hbm.25701

**Published:** 2021-10-29

**Authors:** Tiago Guardia, Linda Geerligs, Kamen A. Tsvetanov, Rong Ye, Karen L. Campbell

**Affiliations:** ^1^ Department of Psychology Brock University St. Catharines Ontario Canada; ^2^ Donders Institute for Brain, Cognition, and Behaviour Radboud University Nijmegen The Netherlands; ^3^ Department of Clinical Neurosciences University of Cambridge Cambridge UK

**Keywords:** ascending arousal system, fMRI, functional networks, neurocognitive aging

## Abstract

A common finding in the aging literature is that of the brain's decreased within‐ and increased between‐network functional connectivity. However, it remains unclear what is causing this shift in network organization with age. Given the essential role of the ascending arousal system (ARAS) in cortical activation and previous findings of disrupted ARAS functioning with age, it is possible that age differences in ARAS functioning contribute to disrupted cortical connectivity. We test this possibility here using resting state fMRI data from over 500 individuals across the lifespan from the Cambridge Center for Aging and Neuroscience (Cam‐CAN) population‐based cohort. Our results show that ARAS‐cortical connectivity declines with age and, consistent with our expectations, significantly mediates some age‐related differences in connectivity within and between association networks (specifically, within the default mode and between the default mode and salience networks). Additionally, connectivity between the ARAS and association networks predicted cognitive performance across several tasks over and above the effects of age and connectivity within the cortical networks themselves. These findings suggest that age differences in cortical connectivity may be driven, at least in part, by altered arousal signals from the brainstem and that ARAS–cortical connectivity relates to cognitive performance with age.

## Graphical abstract

A common finding in the aging literature is that of the brain's decreased within‐ and increased between‐network functional connectivity. However, it remains unclear what is causing this shift in network organization with age. Our findings suggest that age differences in cortical connectivity may be driven, at least in part, by altered arousal signals from the brainstem and that ARAS–cortical connectivity relates to cognitive performance with age.

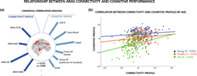

## 1 INTRODUCTION

As people age from young to older adulthood, several changes are commonly observed in the brain's functional network organization, including reduced suppression of the default mode network during task performance, differences in network interactivity, and decreased network segregation (Bethlehem et al., 2020; Chan, Park, Savalia, Petersen, & Wig, 2014; Damoiseaux, 2017; Ferreira et al., 2016; Grady, 2012; Spreng, Wojtowicz, & Grady, 2010; Turner & Spreng, 2015). Age differences in network segregation are characterized by decreased within‐network and increased between‐network functional connectivity from adulthood onwards, meaning that functional networks become less distinct in older age (Chan et al., 2014; Geerligs, Renken, Saliasi, Maurits, & Lorist, 2014). This age‐related decline in network segregation is particularly pronounced amongst association networks [i.e., the default mode network (DMN), salience network (SN), dorsal attention network (DAN), and frontoparietal control network (FPCN)] (Chan et al., 2014; Geerligs et al., 2014) and has been observed in both task and resting state fMRI studies (Spreng, Stevens, Viviano, & Schacter, 2016), when controlling for neurovascular coupling of the BOLD signal (Tsvetanov et al., 2016), and when the brain's electrophysiological signaling is measured directly with EEG (Petti et al., 2016). Moreover, these networks in particular have been consistently related to age‐related declines in general cognitive functioning (Grady, Sarraf, Saverino, & Campbell, 2016; Sala‐Llonch, Bartres‐Faz, & Junque, 2015; Shaw, Schultz, Sperling, & Hedden, 2015; Siman‐Tov et al., 2017). Despite advances in characterizing the effects of age on functional brain networks, a complete understanding of the factors involved in this shifting balance between intra‐network and inter‐network connections is still lacking.

Could one of the factors contributing to altered cortical connectivity be coming from outside the cortex? The ascending arousal system, also known as the ascending reticular activating system (ARAS) is composed of a set of neuroanatomic structures and neurotransmitter systems connecting the brainstem to the cortex and promoting cortical arousal, an essential component of awareness. A diffuse set of neuronal projections from multiple brainstem nuclei stimulate the cerebral cortex via ascending pathways that project to the thalamus, posterior hypothalamus, basal forebrain, and directly to the cortex itself (Jones, 2003). These thalamic and extrathalamic ascending pathways include, but are not limited to, glutamatergic fibers from the parabrachial complex, cholinergic fibers from the pedunculopontine nucleus, noradrenergic fibers from the locus coeruleus, dopaminergic fibers from the ventral tegmental area, and serotonergic fibers from the raphe nuclei (Edlow et al., 2012). This complex set of neurotransmitter pathways that compose the ARAS continuously interact with and modulate one another on route to the cortex, affecting brain functioning and influencing many aspects of cognition (Briand, Gritton, Howe, Young, & Sarter, 2007; Handra et al., 2019; Lobo & Summavielle, 2016).

During the aging process, there is a clear disruption to the ARAS, whereby loss of neurons and receptors is associated with a compensatory increase in neurotransmitter system activity along the ascending pathways (Handra et al., 2019). However, despite recent advances in our understanding of how age affects ARAS functioning (Mather, 2020), neuroimaging studies that investigate the effect of age on functional connectivity of the arousal system are still scarce and restricted to specific nuclei of the system. For instance, in recent years, there has been increased interest in the role of the locus coeruleus (LC) in neurocognitive aging, and structural neuroimaging findings suggest that LC integrity is associated with cognitive reserve and behavioral performance in older adults (Clewett et al., 2016; Dahl et al., 2019; Liu et al., 2020). Recent functional neuroimaging studies have also started to investigate age differences in functional connectivity of brainstem nuclei (Jacobs, Müller‐Ehrenberg, Priovoulos, & Roebroeck, 2018; Serra et al., 2018; Zhang, Hu, Chao, & Li, 2016); but no study to date has examined age differences in functional connectivity across the entire ARAS and determined its relationship to cortical connectivity.

Thus, the first goal of the current study was to examine the whole‐brain connectivity pattern of the brainstem nuclei of the ARAS and age differences therein using resting state fMRI data from the Cambridge Center for Aging and Neuroscience's (Cam‐CAN) population‐based cohort. Previous studies examining age differences in functional connectivity of ARAS nuclei have reported a complex pattern of results, with some connections increasing with age and others decreasing depending on the brain area (Jacobs et al., 2018; Zhang et al., 2016). Thus, we hypothesized that aging would be associated with a diverse set of ARAS–cortical connectivity differences, including both increases and decreases in connectivity to different regions of the brain.

Further, given the essential role of the arousal system in cortical activation and previous findings of disrupted ARAS functioning with age, we hypothesized that age differences in ARAS connectivity relate to concomitant differences in functional network segregation. As already discussed, decreased segregation is characterized by a decrease in within network connectivity and an increase in between network connectivity and is particularly pronounced for the association networks (Chan et al., 2014; Geerligs et al., 2014). Thus, we determined the extent to which age differences in association network connectivity are explained by age‐related declines in ARAS‐association network connectivity.

Finally, we predicted that the multivariate relationship between ARAS‐association network connectivity and cognitive performance will vary with aging (Bethlehem et al., 2020; Tibon et al., 2021; Tsvetanov et al., 2016, 2021). Since the DMN, FPCN, DAN, and SN have primarily been implicated in memory and attentional control (e.g., Grady et al., 2016; Sala‐Llonch et al., 2015; Shaw et al., 2015; Siman‐Tov et al., 2017), we limited our analyses to tasks from the CamCAN that measure these cognitive functions (including ACE‐R, Cattell test of fluid intelligence, Story Recall, Choice Reaction Time, and Visual Short‐term Memory). To this end, we ran a canonical correlation analysis relating ARAS‐association network connectivity measures to our cognitive variables of interest.

## 2 METHODS

### 2.1 Participants

A sample of 644 participants (18–88 years old; mean 54.2; *SD* 18.5; 319 males and 325 females) was taken from the population‐derived Stage 2 sample of the Cambridge Center for Aging and Neuroscience (Cam‐CAN) project (Shafto et al., 2014). After excluding participants based on motion correction and cardiovascular health (described further below), a final sample of 535 participants (18–88 years old; mean 53.9; *SD* 17.5; 272 males and 263 females) approximately equally distributed across the lifespan remained. Demographic information of the current sample is provided in Table 1 (divided into age groups for illustrative purposes, but all analyses used age as a continuous variable). Participants were included if they had no contraindications to MRI, no self‐reported history of drug or alcohol abuse, no neurological disorders, and no brain abnormalities detected. Participants were native English speakers, had normal or corrected‐to‐normal vision and hearing, and scored 25 or higher on the mini mental state exam (MMSE). Informed consent was obtained from all participants and the study was approved by the Cambridgeshire 2 Research Ethics Committee, United Kingdom (Shafto et al., 2014).

**TABLE 1:** Participant demographics and cognitive scores

Age group	Young	Middle	Older	Total
*n*	194	178	163	535
Age range (years)	18–45	46–64	65–88	18–88
Sex (male/female)	104/90	85/93	83/80	272/263
*Highest education*				
University	147 (75.8%)	117 (65.7%)	58 (32.6%)	322 (60.2%)
A' levels	26 (13.4%)	35 (19.7%)	58 (32.6%)	119 (22.2%)
GCSE grade	19 (9.8%)	19 (10.7%)	24 (13.5%)	62 (11.6%)
None over 16	1 (0.5%)	7 (3.9%)	22 (12.4%)	30 (5.6%)
*Cognitive scores*				
MMSE	29.32 (1.05)	29.17 (1.05)	28.42 (1.36)	28.99 (1.2)
ACE‐R	96.48 (3.42)	96.00 (3.52)	92.76 (5.20)	95.19 (4.38)

*Note*: Education data missing for two participants. ACE‐R and MMSE scores missing for two participants.

### 2.2 Image acquisition

Participants were instructed to rest with eyes closed and to not think of anything in particular during fMRI scanning. Scanning took place at the Medical Research Council Cognition and Brain Sciences Unit (MRC‐CBSU) in a 3 T Siemens TIM Trio, with a 32‐channel head‐coil. For resting state, 261 volumes were acquired, each containing 32 axial slices (acquired in descending order), with slice thickness of 3.7 mm and interslice gap of 20% (for whole‐brain coverage including cerebellum; TR 1970 ms; TE 30 ms; flip angle 78°; FOV 192 mm × 192 mm; voxel size 3 mm × 3 mm × 4.44 mm and acquisition time of 8 min and 40 s. Higher‐resolution (1 mm × 1 mm × 1 mm) T1 and T2 weighted structural images were also acquired to aid registration across participants (Shafto et al., 2014; Taylor et al., 2017).

### 2.3 Data preprocessing

**FIGURE 1:**
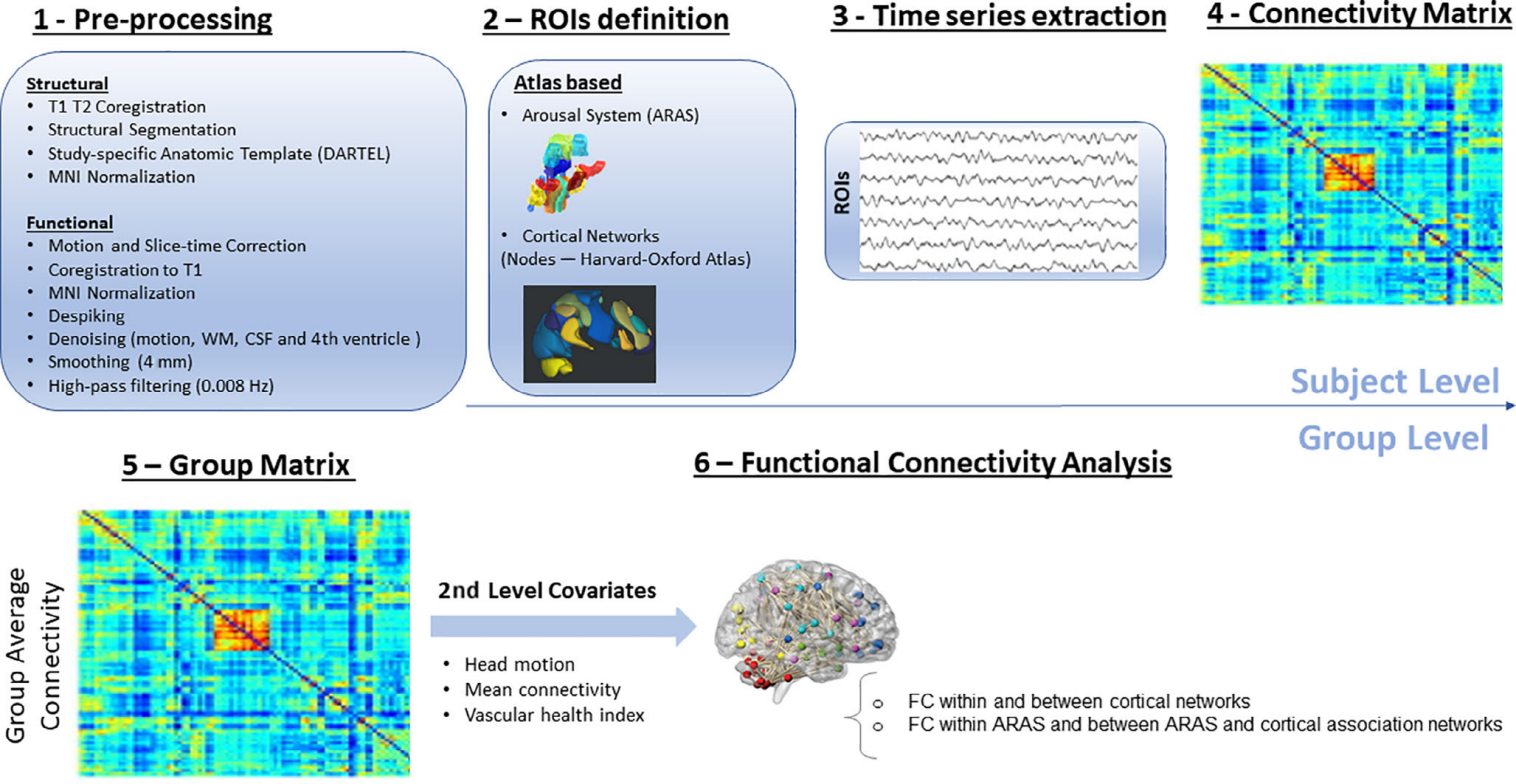
fMRI data preprocessing and analysis pipeline. Nodes of canonical cortical networks (Harvard–Oxford atlas) encompasses the default mode network (DMN), dorsal attention network (DAN), frontoparietal control network (FPCN), and salience network (SN). Additional networks included in the whole‐brain connectivity analysis (i.e., canonical cortical networks) included the sensorimotor, visual, language, and cerebellum. The arousal system nuclei included the dorsal raphe nucleus (DR), mesencephalic reticular formation (MRF), median raphe nucleus (MR), periaqueductal gray (PAG), parabrachial complex (PBC), pontine nucleus oralis (PO), pedunculopontine tegmental nucleus (PPN), ventral tegmental area (VTA), and locus coeruleus (LC)

An overview of the analysis pipeline is shown in Figure 1. Using SPM 12 software (http://www.fil.ion.ucl.ac.uk/spm) and the automatic analysis (AA) batching system (http://imaging.mrc-cbu.cam.ac.uk/imaging/), T1 and T2 structural images were coregistered. Unified segmentation was performed on the combined images (Ashburner & Friston, 2005) and subsequently, the gray matter (GM) and white matter (WM) segments of each participant were used to create a study‐specific anatomical template using the DARTEL procedure to optimize interparticipant alignment (Ashburner, 2007), which was subsequently normalized into MNI space. For each participant, field maps were used to undistort the functional EPI T2* images and then the functional images were motion‐corrected and slice‐time corrected. Subsequently, the EPI images were coregistered to the T1 image and the DARTEL flow fields were applied for MNI normalization (Taylor et al., 2017).

To reduce the effects of motion on our measures of functional connectivity (e.g., Power, Barnes, Snyder, Schlaggar, & Petersen, 2012; Satterthwaite et al., 2012) additional motion correction procedures were applied. The first step was to apply a wavelet despike method for removing motion artifacts from fMRI data without the need for data scrubbing (Patel et al., 2014). Participants with an average spike percentage of two *SD*s above the mean were excluded from further analysis (this led to the exclusion of 58 participants). The second step to reduce the effects of motion and other noise confounds was to apply multiple regression of the six original motion parameters as well as average signals from white matter (WM), and cerebrospinal fluid (CSF). We also regressed out the signal of the fourth ventricle due to its proximity to the nuclei of the brainstem (Ngeles Fernández‐Gil, Palacios‐Bote, Leo‐Barahona, & Mora‐Encinas, 2010). Additionally, in order to reduce the confounding effects of head motion and vascular health, for each participant, mean connectivity across all connections was calculated and regressed out of subsequent analyses at the group level (Geerligs, Tsvetanov, Cam‐CAN, & Henson, 2017). This method has been shown to increase the reliability of both connectivity estimates and effects of age while simultaneously reducing associations between connectivity and vascular health, and between connectivity and head motion (Geerligs et al., 2017).

Next, a high‐pass filter of 0.008 Hz was implemented. Although band‐pass filtering is commonly used to reduce physiological noise, it also leads to less reliable estimates of functional connectivity (Shirer, Jiang, Price, Ng, & Greicius, 2015) and research has shown that the effect of age is better identified when applying high‐pass than band‐pass filtering (Geerligs et al., 2017).

Age is also associated with changes in cardiovascular health and neurovascular coupling, which is known to affect the BOLD signal and measures of functional connectivity (Abdelkarim et al., 2019; D'Esposito, Deouell, & Gazzaley, 2003; Hutchison, Lu, & Rypma, 2013; Hutchison, Shokri‐Kojori, Lu, & Rypma, 2013; Tsvetanov et al., 2015; Tsvetanov, Henson, & Rowe, 2020). In order to minimize these effects, participants with cardiovascular disease (65 individuals) were excluded from the current sample and we controlled for vascular function in the rest of the sample by regressing out a composite measure of vascular health (Tsvetanov et al., 2015). This vascular health index was obtained by taking the first principal component from a principal components analysis (PCA) applied to a number of heart rate measures obtained using photoplethysmography/pulseoximeter during scanning. These included: mean heart rate (HR), low‐frequency heart rate variability (LF‐HRV; 0.05–0.15 Hz), and high‐frequency heart rate variability (HF‐HRV; 0.15–0.4 Hz). The PCA analysis estimated the first principal component (PC1) to explain 67.96% of the variance across the three summary measures of HR.

### 2.4 Data analysis

#### 2.4.1 ARAS and cortical network connectivity

Regions of interest (ROIs) for our functional connectivity analysis included the ARAS structures from the Harvard Ascending Arousal Network Atlas (Martinos Center for Biomedical Imaging, Charleston, Massachusetts; https://www.martinos.org/resources/aan-atlas) (Edlow et al., 2012): dorsal raphe nucleus (DR), mesencephalic reticular formation (MRF), median raphe nucleus (MR), periaqueductal gray (PAG), parabrachial complex (PBC), pontine nucleus oralis (PO), pedunculopontine tegmental nucleus (PPN), and ventral tegmental area (VTA). For the locus coeruleus (LC) ROI, we used a LC probabilistic atlas developed by using ultrahigh field 7 T MRI (Ye et al., 2021). ROI masks of nodes from canonical brain networks [default mode (DMN)—four nodes, salience (SN)—seven nodes, dorsal attention (DAN)—four nodes, frontoparietal control (FPCN)—four nodes, sensorimotor (SM)—three nodes, visual (VIS)—four nodes, language (LAN)—four nodes, and cerebellar (CEREB)—two nodes] were taken from the FSL Harvard‐Oxford atlas available in the Conn Toolbox v.18b (https://web.conn-toolbox.org/). For each subject, the Pearson's correlation coefficients were calculated between the preprocessed fMRI time series of each ROI and the time courses of all other ROIs and transformed to *Z*‐values using the Fisher transformation (Bianciardi et al., 2016). These *Z*‐values were then used in second‐level group analyses to assess (a) mean connectivity within the ARAS, and between the ARAS and other brain networks, (b) mean connectivity within and between cortical networks, and (c) the effect of age on ARAS and cortical network connectivity (false discovery rate [FDR] corrected two‐sided *p*‐value <.05; Figure 1). Group level analyses were all performed by controlling for mean connectivity across all ROIs, head motion, vascular health index, and education level (Chan et al., 2014).

#### 2.4.2 Within/between network connectivity analyses

**FIGURE 2:**
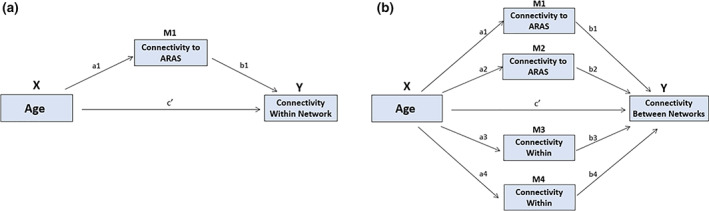
Mediation analyses looking at the effect of ARAS‐association network connectivity on the age‐related decline in functional connectivity within and between the association networks (averaged across nodes within each network/module). (a) Depiction of the within‐network connectivity mediation model. (b) Depiction of the between‐network connectivity mediation model. Mediators M1 and M2 are the connectivity of each module to the arousal system

The association between the arousal system and connectivity within and between cortical networks was assessed specifically for the association networks (i.e., DMN, SN, DAN, and FPCN), as age differences in segregation are typically strongest for these networks (Chan et al., 2014; Geerligs et al., 2014). The aim was to evaluate the percentage of age‐related variance in functional connectivity of the association networks that is shared with age‐related differences in ARAS–cortex connectivity. This was achieved by performing a series of mediation analyses to test the mediating effect of the mean connectivity across all arousal system nuclei and each pair of networks (within network connectivity: DMN, SN, DAN, and FPCN; Figure 2a; between network connectivity: DMN × SN, DMN × DAN, DMN × FPCN, SN × DAN, SN × FPCN, and DAN × FPCN; Figure 2b).

In this study, we aim to assess the effects of the ARAS as an integrated system, thus, instead of analyzing the mediation effect of each brainstem nucleus separately, the average connectivity between all ARAS ROIS and the ROIs in a given cortical network was used as a representative metric of the connectivity between the ARAS and that network as a whole. Thus, for each analysis, we aimed to explain the direct effect of age on functional connectivity within a network or between a pair of networks. In the case of within network connectivity, the connectivity between that network and the arousal system (averaged across all ARAS ROIs) was used as a mediator, so only one mediator (see Figure 2a). For between network connectivity, the average connectivity between the ARAS nuclei and both functional networks being analyzed were used as mediators, so two mediators (see Figure 2b). These analyses were run in the Mediation Analysis Toolbox in Matlab using bootstrapping with 10,000 samples and FDR was used for the correction of multiple comparisons (Wager, Davidson, Hughes, Lindquist, & Ochsner, 2008).

#### 2.4.3 Relationship between ARAS–cortical connectivity and cognitive performance

For the connectivity‐behavior analysis, we adopted a two‐level approach (Passamonti et al., 2019; Tibon et al., 2021; Tsvetanov et al., 2016; 2021). In the first level, to determine which (if any) connections between the ARAS and cortex were important for cognitive performance, we ran a canonical correlation analysis (CCA; Sui, Adali, Yu, Chen, & Calhoun, 2012; Wang et al., 2020) to identify linear relationships between the two sets of measures (ARAS‐association network connectivity, and cognitive performance in our main cognitive variables of interest from Cam‐CAN). The set of cognitive variables included the following cognitive tasks: Addenbrooke's Cognitive Examination‐revised (ACE‐R), Cattell Culture Fair test of fluid intelligence, Story Recall, Choice Reaction Time, and Visual Short‐term Memory (full descriptions of the tasks are available in Appendix S1). The first step was to run CCA on both sets of variables (Set 1, ARAS Connectivity; Set2, Cognitive Performance), through which linear combinations within each of the sets were determined in a way that the relationship of the combinations between both sets was maximized. This resulted in a pair of significantly correlated canonical variates (i.e., latent variables), which we refer to as X1—connectivity subject scores, and Y1—cognitive subject scores.

Next, we tested whether the relationship between ARAS connectivity and cognitive performance varies with age. To this end, we performed a second‐level analysis using moderation analysis. In our model, ARAS connectivity subject scores, age, their interaction term (ARAS connectivity subject scores × age), and covariates of no interest [mean connectivity across all ROIs, head motion, vascular health index, mean connectivity within each of the association networks (i.e., DMN, SN, DAN, and FPCN within‐network connectivity), and education level] were used as independent variables and cognitive subject scores as a dependent variable.

## 3 RESULTS

### 3.1 ARAS and cortical network connectivity

**FIGURE 3:**
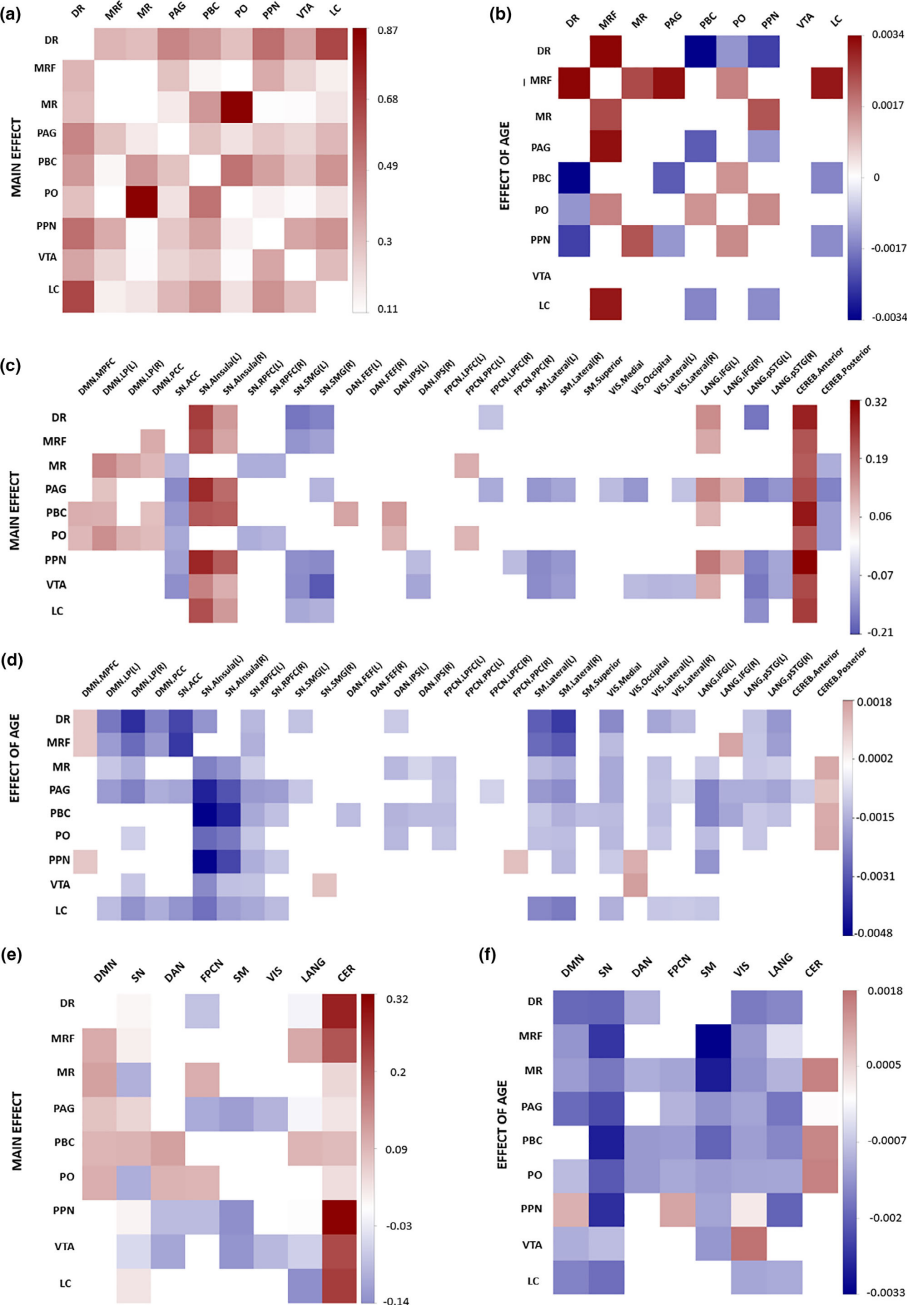
Functional connectivity analysis of the arousal system's nuclei (controlling for mean connectivity across all ROIs, head motion, WM, CSF, fourth ventricle, vascular health index and education level). Correlation matrices of functional connectivity (Pearson *r*, *p*‐FDR < .05) for (a) main effect—Connectivity within ARAS; (b) effect of age—connectivity within ARAS; (c) main effect—connectivity between ARAS and cortical networks; (d) effect of age—connectivity between ARAS and cortical networks; (e) main effect—connectivity between ARAS and cortical networks (average per network); (f) effect of age—connectivity between ARAS and cortical networks (average per network). Functional modules included in the analysis: default mode network (DMN), salience network (SN), dorsal attention network (DAN), frontoparietal control network (FPCN), sensorimotor network (SM), visual network (VIS), language network (LAN), and cerebellar network (CEREB). The arousal system nuclei included in the analysis are: dorsal raphe nucleus (DR), mesencephalic reticular formation (MRF), median raphe nucleus (MR), periaqueductal gray (PAG), parabrachial complex (PBC), pontine nucleus oralis (PO), pedunculopontine tegmental nucleus (PPN), ventral tegmental area (VTA), and locus coeruleus (LC)

The ARAS nuclei were all positively correlated with each other (see Figure 3a) and widely connected to several cortical networks (Figure 3c,e). Specifically, ARAS nuclei showed several positive connections to the default mode network, insula, and inferior frontal gyrus and negative connections to other regions of the salience network (apart from the insula) and the visual network.

Within the ARAS, age was associated with both increases and decreases in connectivity (see Figures 3b). In contrast, ARAS–cortical connectivity was largely characterized by an age‐related decrease in the number and strength of positive connections (particularly between the ARAS and some nodes of the default mode and salience networks), but also an increase in the strength of some anti‐correlations (Figure 3d,f).

We also examined functional connectivity within/between four cortical association networks previously shown to become less segregated with age (i.e., DMN, SN, DAN, FPCN—see Section 2). In line with previous work, nodes from each network clustered together, with most showing higher intrinsic and lower extrinsic connectivity (except for SN to DAN; see Figure S1A). Further, in line with previous findings, age was largely associated with decreased within‐network (*r* = −.172; *p* < .001) and increased between‐network (*r* = .155; *p* < .001) functional connectivity (Figure S1B).

### 3.2 Mediation effects of ARAS–cortical connectivity on age differences in cortical connectivity

To determine whether some of the age‐related variance in functional connectivity within and between the association networks is shared with age‐related differences in ARAS–cortex connectivity, we performed a series of mediation analyses (see Figure 2 and Methods; all analyses FDR corrected for multiple comparisons). Our results show that age is associated with a general decline in connectivity between the ARAS and association networks [Tables S1 and S2 (a paths)], and that this partly explains some of the observed age‐related differences in association network connectivity. Specifically, we found that connectivity between the ARAS and DMN partly explains the age‐related decline in connectivity within the DMN (*b* = −0.03, 95% CI [−0.03, −0.02], *t* = −2.54, *p* = .002) and the age‐related increase in connectivity between the DMN and SN (*b* = 0.02, 95% CI [0.01, 0.03], *t* = 2.09, *p* = .005). Additionally, we also found two effects that were significant at *p* < .05, but did not survive FDR correction: ARAS–DMN connectivity was associated with the age‐related increase in connectivity between the DMN and DAN (*b* = 0.01, 95% CI [0.01, 0.02], *t* = 1.75, *p* = 0.031), and ARAS–SN connectivity was associated with the age‐related decrease in connectivity between the SN and DAN (*b* = −0.03, 95% CI [−0.03, −0.02], *t* = −2.03, *p* = 0.024). Table 2 summarizes the list of models and significant effects (see Tables S1 and S2 for complete results of each model; Tables S3 and S4 show the effects of each nucleus separately for the significant network‐level mediation effects).

**TABLE 2:** List of mediation models and significant mediation effects

	Model	X	Y	Mediator	*p*
Within‐network connectivity models	1	AGE	DMN	ARAS–DMN	**.002
	2	AGE	SN	ARAS–SN	.309
	3	AGE	DAN	ARAS–DAN	.378
	4	AGE	FPCN	ARAS–FPCN	.608
Between‐network connectivity models	5	AGE	DMN–SN	ARAS–DMN	**.005
		AGE	DMN–SN	ARAS–SN	.367
	6	AGE	DMN–DAN	ARAS–DMN	*.031
		AGE	DMN–DAN	ARAS–DAN	.455
	7	AGE	DMN–FPCN	ARAS–DMN	.391
		AGE	DMN–FPCN	ARAS–FPCN	.296
	8	AGE	SN–DAN	ARAS–SN	*.024
		AGE	SN–DAN	ARAS–DAN	.630
	9	AGE	SN–FPCN	ARAS–SN	.053
		AGE	SN–FPCN	ARAS–FPCN	.142
	10	AGE	DAN–FPCN	ARAS–DAN	.769
		AGE	DAN–FPCN	ARAS–FPCN	.126

*Note*: *significant at *p* < .05; **significant after FDR correction.

### 3.3 Relationship between ARAS–cortical connectivity and cognitive performance

**FIGURE 4:**
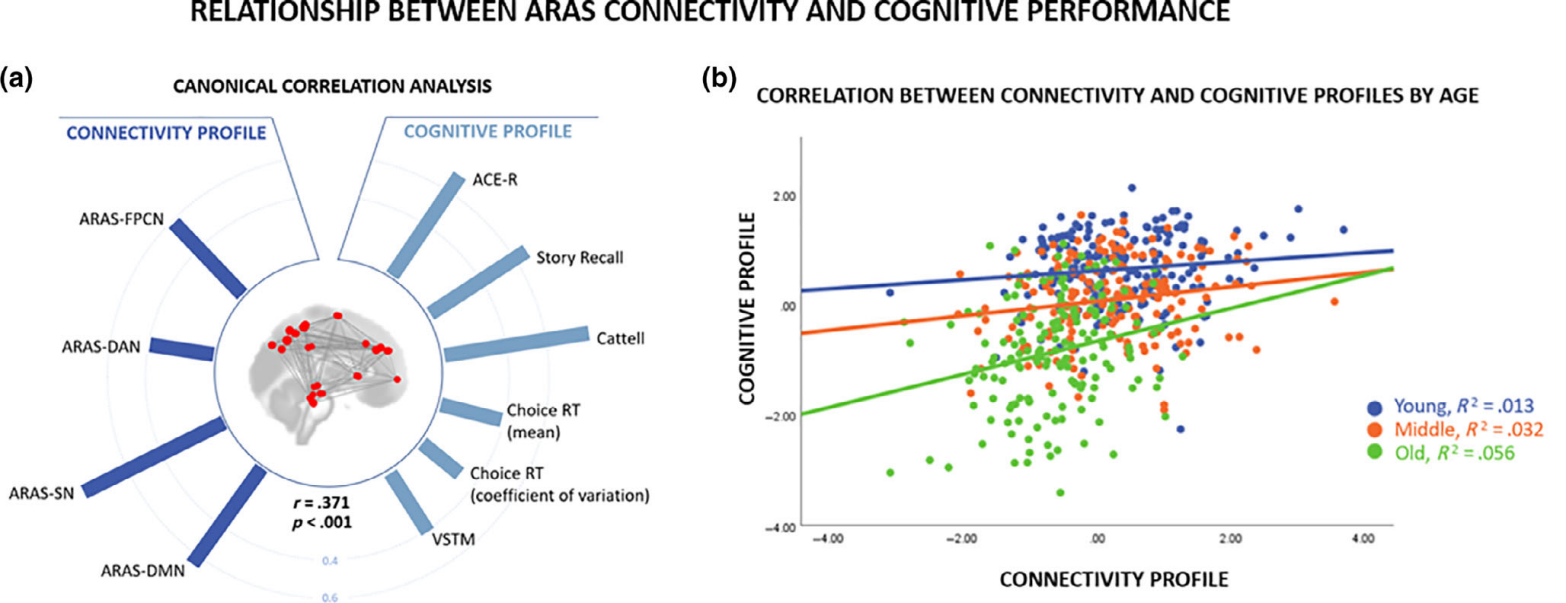
The relationship between ARAS connectivity and cognitive performance. (a) Canonical correlation analysis (CCA). Heliograph of variate loadings (correlations) for the first canonical variate, where the relative size of the correlations is indicated by the relative length of the bars. The statistical relationship between variables of functional connectivity (connectivity profile) and cognitive performance (cognitive profile) is *r* = .371, *p* < .001. The direction of the Choice RT variables is flipped, so that higher scores reflect faster and less variable responding (i.e., better performance). (b) The relationship between connectivity and cognitive profiles by age‐group. Higher subject loading values indicate stronger expression of the cognitive and connectivity profiles, and the relationship between these is moderated by age (see Table 3)

To determine whether connectivity between the ARAS and association networks relates to cognitive functioning, we ran a canonical correlation analysis to identify relationships between ARAS‐association network connectivity and cognitive performance across a range of tasks. The first canonical vector was significant (*r* = .371; *p* < .001) and identified that higher levels of ARAS‐association network connectivity (ARAS–DMN, ARAS–SN, ARAS–DAN, and ARAS–FPCN) was associated with better levels of performance in all the cognitive tasks (see Figure 4a). To further investigate the relationship between cognitive performance and ARAS connectivity profiles, we conducted a moderation analysis including ARAS connectivity subject scores, age, their interaction term (connectivity subject scores × age), and covariates of no interest [mean connectivity across all ROIs, head motion, vascular health index, mean connectivity within each of the association networks(i.e., DMN, SN, DAN, and FPCN within‐network connectivity), and education level] as independent variables and cognitive subject scores as a dependent variable. The results are shown in Table 3. The interaction term between connectivity profile and age predicted variance in cognitive performance, (*b* = 0.081, *p* = .01), and the direction of the interaction was such that increasing age strengthened the relationship between ARAS connectivity and cognitive performance profiles. It is worth noting that age was a continuous variable in the analysis, although for clarity of illustration in Figure 4b, we divide the cohort into young (18–45 years) middle (45–64 years) and older age groups (65–78 years).

**TABLE 3:** Moderation analysis

Outcome	Predictors	*b*	*SE*	*t*	Sig.
Cognitive performance profile	Connectivity profile	0.123	0.037	3.343	<.001
	Age	−0.463	0.045	−10.345	<.001
	Connectivity profile × age	0.081	0.031	2.593	.01

*Note*: Predictors: Connectivity profile, age, cognitive profile × age, DMN‐DMN, SN‐SN, DAN‐DAN, FPCN‐FPCN, head motion, cardiovascular health index, mean connectivity.

## 4 DISCUSSION

The main goals of this study were to (a) characterize functional connectivity patterns of all brainstem arousal system nuclei and age differences therein, (b) test whether age differences in connectivity within and between association networks relate to age differences in ARAS‐association network connectivity, and (c) evaluate the relationship between ARAS–cortical connectivity and cognitive performance. Our results show that the ARAS nuclei have positive intrinsic connections, as well as positive and negative connections to the cortex, particularly the default mode and salience networks. In general, aging was associated with reduced ARAS‐association network connectivity and, replicating previous work, decreased within‐network and increased between‐network functional connectivity in the cortex. Additionally, consistent with our predictions, we found that functional connectivity between the arousal system and association networks was significantly associated with age‐related differences in connectivity within and between association networks, suggesting that age differences in ARAS functioning may contribute to altered cortical connectivity with age. Finally, we also found that higher connectivity between the ARAS association networks relates to better cognitive performance, and that this relationship is moderate by age.

### 4.1 ARAS connectivity and the effects of aging

Advances in neuroimaging have allowed for the investigation of arousal system functioning in humans and its effects on cortical activity and cognition (Beissner, Schumann, Brunn, Eisenträger, & Bär, 2014; Sclocco, Beissner, Bianciardi, Polimeni, & Napadow, 2018). Previous work has shown that the dopaminergic ventral tegmental area (VTA) and the serotonergic dorsal raphe nucleus (DR) are functionally connected to the default mode network, and that the noradrenergic locus coeruleus is connected to the frontoparietal network (Bär et al., 2016). Here, we characterized functional connectivity of the entire ARAS, showing that ARAS nuclei are positively connected to one another and to nodes of the DMN, SN, DAN, FPCN, language, and cerebellar networks. Some ARAS nuclei were negatively correlated with nodes of the SN, DAN, sensorimotor, visual, language, and cerebellar networks. These findings are in line with previous studies showing that ARAS nuclei are widely connected with cortical regions (Bär et al., 2016; Bianciardi et al., 2016; Englot et al., 2017; Parra‐Morales et al., 2019). Previous work has shown positive connectivity between the DMN and the ventral tegmental area (VTA), dorsal raphe (DR), media raphe (MR), and periaqueductal gray area (PAG) (Bär et al., 2016; Bianciardi et al., 2016). In addition to these nuclei, we also found positive connectivity between the DMN and the mesencephalic reticular formation (MRF), parabrachial complex (PBC), pontine nucleus oralis (PO), and locus coeruleus (LC). Additionally, our results show positive connectivity between the salience network (SN) and LC and DR, which is similar (though not identical) to previous work showing connectivity between these nuclei and the frontoparietal network (Bär et al., 2016; Bianciardi et al., 2016).

We also examined the effects of age on ARAS connectivity. Within the ARAS itself, we found that some connections increased with age, while others decreased. In contrast, the effect of age on ARAS–cortical connectivity mainly consisted of an increase in negative connections and a reduction in positive connections, which was particularly pronounced for connections to the DMN and SN. Further, the locus coeruleus, periaqueductal gray, and dorsal raphe nucleus appeared to show the strongest age‐related decline in connectivity to the cortex, which could reflect age‐related physiological disruptions in the noradrenergic and serotonergic neurotransmitter systems (Mather, 2020).

While our aging results largely align with previous work (Bär et al., 2016; Bianciardi et al., 2016; Englot et al., 2017; Parra‐Morales et al., 2019), some differences are apparent. For instance, previous work has found both non‐linear and positive effects of age on connectivity between the LC and cortical networks (Jacobs et al., 2018; Zhang et al., 2016), while in our study, we only found a negative effect of age on LC–cortical connectivity. These discrepancies may be due to methodological differences. In our study, we used high‐pass filtering and controlled for vascular health, motion, and mean connectivity across the whole brain. These pre‐processing strategies have been shown to lead to more reliable estimates of age‐differences in connectivity that are less affected by confounds such as vascular health and head motion (Geerligs et al., 2017), and therefore might be the reason why we found some divergences with previous findings of the literature.

### 4.2 Mediation effects of the ARAS on age differences in cortical connectivity

The ARAS plays a critical role in cortical activation (Aston‐Jones, 2005; Edlow et al., 2012; Jones, 2003) and shows marked changes with age (Jacobs et al., 2018; Lee, Kim, Katz, & Mather, 2020; Mather, 2020; Zhang et al., 2016); thus, we hypothesized that age differences in ARAS‐association network connectivity may be associated with commonly observed age differences in association network connectivity. We found that decreased ARAS–DMN connectivity with age partly explained the age‐related decline in connectivity within the DMN. Age‐related declines in DMN connectivity are commonly observed at rest (Grady, Springer, Hongwanishkul, McIntosh, & Winocur, 2006; Hafkemeijer, van der Grond, & Rombouts, 2012; Sambataro et al., 2010) and these results suggest that reduced arousal modulation of the DMN may be associated with this decline. Relatedly, several studies have reported that noradrenergic, dopaminergic, and serotonergic pharmacological interventions affect DMN connectivity (van den Brink, Pfeffer, & Donner, 2019), lending support to the idea that age‐related changes in brainstem nuclei functioning may contribute to age differences in DMN connectivity. We also found that decreased ARAS–DMN connectivity with age partly explained age‐related differences in DMN–SN connectivity. The salience network is thought to be responsible for processing salient stimuli in the environment and modulating the switch between internally oriented cognitive processes of the DMN and externally oriented cognitive processes of the task positive networks (He et al., 2013; Sridharan, Levitin, & Menon, 2008; Uddin, 2015). Altered connectivity between the ARAS and DMN with age and its impact on DMN–SN connectivity may have knock‐on effects, affecting the switch between internally oriented and externally oriented attention.

Anatomically, the ARAS nuclei have widespread projections to cortical regions, synthesizing and releasing modulatory neurotransmitters that affect neural activity across the cortex. Many studies have shown that pharmacological interventions on these neurotransmitter systems result in diverse changes in cortical network states, by altering the strength and topography of functional connectivity (van den Brink et al., 2019). Further, dynamic analyses of fMRI data have demonstrated that cortical networks transition between segregated and integrated states within the duration of a typical scan (Shine et al., 2016). Importantly, integrated network states correlate with increases in pupil diameter (a biomarker of arousal), suggesting some role of the ARAS system in cortical connectivity in line with the current results (Shine et al., 2016).

The aging process is associated with structural degeneration and functional disruptions in the arousal system, which is thought to contribute to age differences in circadian and sleep–wake regulation (Mander, Winer, & Walker, 2017). However, age‐related deterioration of the ARAS is thought to be compensated for by increased levels of some neurotransmitters, which may help older adults maintain sufficient levels of alertness and cognitive functioning (Mather, 2020). As already discussed, pharmacological interventions on these neurotransmitter systems can affect cortical network topology, including measures of network integration (Achard & Bullmore, 2007; Schaefer et al., 2014; Shine, van den Brink, Hernaus, Nieuwenhuis, & Poldrack, 2018; van den Brink et al., 2016). It is possible that age‐related compensatory increases in activity of some of the neurotransmitters systems (e.g., noradrenergic and dopaminergic; Mather, 2020) is one of the mechanisms by which aging affects the balance between intranetwork and internetwork connectivity, though this hypothesis requires further investigation.

### 4.3 Relationship between ARAS–cortical connectivity and cognition

We tested the multivariate association between ARAS‐association network connectivity and age‐related changes in cognitive performance given that maintaining cortical‐wide connectivity is increasingly important for performance in old age (Bethlehem et al., 2020; Tibon et al., 2021; Tsvetanov et al., 2016; Tsvetanov et al., 2021). Overall, higher levels of ARAS‐association network connectivity were associated with better levels of cognition. The ARAS is comprised of a complex set of neurotransmitter pathways that affect brain functioning and influence cognition, and previous research has demonstrated that changes in those neurotransmitter systems impact working and episodic memory, processing of salient stimuli, and executive functions (Briand et al., 2007; Handra et al., 2019; Lobo & Summavielle, 2016). In our study, we found that connectivity between the ARAS and DMN, SN, DAN, and FPCN networks is associated with better performance across a range of cognitive tasks largely measuring memory and attention, thereby supporting the hypothesis that in addition to cortical activation, the ARAS might play a role in cognition.

Our findings from the moderation analysis suggested that maintaining youth‐like ARAS‐association network connectivity becomes progressively more important for maintaining cognitive functioning in old age. This is consistent with previous findings based on neuronal signatures of cortical connectivity from magnetoencephalography data (Bruffaerts et al., 2019; Tibon et al., 2021) or hemodynamic signatures from fMRI BOLD data only after controlling for physiological and vascular confounds (Bethlehem et al., 2020; Geerligs et al., 2017; Tsvetanov et al., 2015). Our study extends findings from previous research by implicating the increasing reliance on ARAS‐association network interactivity to sustain cognitive performance with increasing age. We propose that preventative and interventional strategies that target such connectivity, possibly via subcortical neuromodulation systems, will promote the well‐being of individuals in old age (e.g., MacInnes, Dickerson, Chen, & Adcock, 2016).

## 5 FINAL CONSIDERATIONS

In this study we showed the arousal system is functionally connected to widespread cortical regions and mediates age‐related differences in cortical networks. However, this study is not without limitations. First, we recognize that our cross‐sectional design is not ideal for capturing the true effects of age. Relatedly, significant mediation effects based on cross‐sectional data cannot be used to infer causality; thus, our results should be interpreted with caution. Changes in cortical connectivity associated with age could also be affecting age‐related changes in ARAS–cortical connectivity. Another limitation is that the ARAS nuclei are small structures located in regions very susceptible to physiological noise. We included a number of procedures to control for motion and reduce physiological noise, but improved signal definition from these small ARAS nuclei [e.g., by using a high‐resolution probabilistic atlas (Ye et al., 2021) may further minimize bias and residual artifacts]. Finally, we examined the effects of ARAS connectivity at rest; however, it has been suggested that resting state data provide a very limited picture of age differences in neurocognitive functioning (Campbell & Schacter, 2017a, 2017b). Future studies should evaluate the influence of the ARAS system by integrating data from both resting state and cognitive tasks (Geerligs & Tsvetanov, 2017).

Age differences in cortical connectivity are now well documented, but we still lack a thorough understanding of the mechanisms underlying those differences. Technological and methodological innovations in data acquisition and analysis are allowing for non‐invasive studies of ARAS functioning in humans. The results of this study and others suggest that the ARAS might play a critical role in determining age differences in the cortex and cognitive health.

## CONFLICT OF INTERESTS

The authors declare no conflicts of interest.

## Supporting information


**Appendix S1**: Supporting Information

## Data Availability

The data that support the findings of this study are available on request from the corresponding authors.
