# Supplementary material for: The role of the arousal system in age‐related differences in cortical functional network architecture
Source: Hum Brain Mapp. 2021 Oct 29;43(3):985–97. doi: 10.1002/hbm.25701 (PMC8764482; doi:10.1002/hbm.25701)
Supplement: Supplementary file 1 — Appendix S1: Supporting Information [file HBM-43-985-s001.docx]

**Supplementary Material**

**METHODS**

Cognitive tasks used in the canonical correlation analysis relating ARAS-cortical connectivity to cognitive performance.

***Addenbrooke’s Cognitive Examination-revised (ACE-R):*** ACE-R (Mioshi et al., 2006) is a brief neuropsychological test that measures general cognitive ability across five domains (attention/orientation, memory, fluency, language, and visuospatial ability). While similar to the Mini-Mental State Exam (MMSE), the ACE-R is more extensive and has been shown to be a more sensitive measure of cognitive ability (Law et al., 2013; Pendlebury et al., 2012; Rittman et al., 2013). In our analysis we used the ACE-R total score, which has a maximum value of 100, and is calculated by the addition of all subtests.

***Cattell Culture Fair test of fluid intelligence:*** Participants completed the standard form of the Cattell Culture Fair, Scale 2 (Cattell, 1971). This paper and pencil test consists of four subtests with different types of nonverbal puzzles: series completion, classification, matrices, and conditions. Before each subtest, instructions and examples are given. Each subtest is timed (3 minutes for the first subtest, 4 minutes for the second, 3 minutes for the third, and 2.5 minutes for the final subtest) although participants are not informed about precise timings beforehand. Correct responses are scored as 1, and the maximum score is 46.

**Story Recall:** We used the Logical Memory subtest of the Weschler Memory Scale Third UK edition (WMS-III-UK) (Wechsler, 1997). Participants are given an oral presentation of a narrative story followed by a delayed (30 min) recall test. The scoring considers the number of details and general thematic ideas recalled from the story, with a maximum score of 25.

**Choice Response Time (RT) Task**: The Choice RT task assesses response speed (Trueman et al., 2012). Participants are shown an image of a hand with blank circles above each of the fingers, while keeping their right hand on a response console with four buttons, one for each finger. When any one of the circles above the fingers becomes black, the participant must press the corresponding finger as quickly as possible (3 seconds RT maximum). On pressing the button (or after 3 seconds), the circle returns to the blank state, and the variable inter-trial interval (ITI) begins. The ITI varies pseudo-randomly (minimum 1.8 seconds, mean 3.7 seconds, and maximum 6.8 seconds). There were 67 trials, and the main outcome is reaction time from stimulus onset to button press. In our study, we used mean RT (averaged across the four fingers - RT values greater than 3 SD from the mean were trimmed) and the coefficient of variation (a common measure of RT variability and one’s ability to stay on-task, calculated as SD/mean for each participant) (Hultsch et al., 2008; MacDonald et al., 2008).

**Visual Short-term Memory:** In this task, participants are shown 1 to 4 colored circular discs to be remembered. After a delay, one of the locations is cued and participants are to report the color of the disc from that location by selecting it from a color wheel with a rainbow of hues (Shafto et al., 2014). On each trial, a central fixation cross and the memory array (set sizes 1 to 4, with the colors chosen at random) were displayed for 250 milliseconds. The locations of the discs on the screen were randomly selected. Following the encoding display, a 900-millisecond blank screen was shown, and then one of the disc locations was highlighted with a border (test display). Participants used a touch screen to indicate the color of the probed item on the color wheel and indicate their confidence in the selected color. Participants had unlimited time to make their response. The task consisted of two blocks of 112 trials each, and estimated parameters include VSTM capacity (K), the accuracy of the reported hues (precision), and the probability of mistakenly reporting an un-cued item. For the current study, we used average capacity (K) across set sizes 3 and 4, as this helped to avoid floor effects in the older group (seen at set size 4) and ceiling effects in the young (seen at set size 3). Set sizes 1 and 2 were too easy and most participants were at ceiling in these conditions.

**RESULTS**


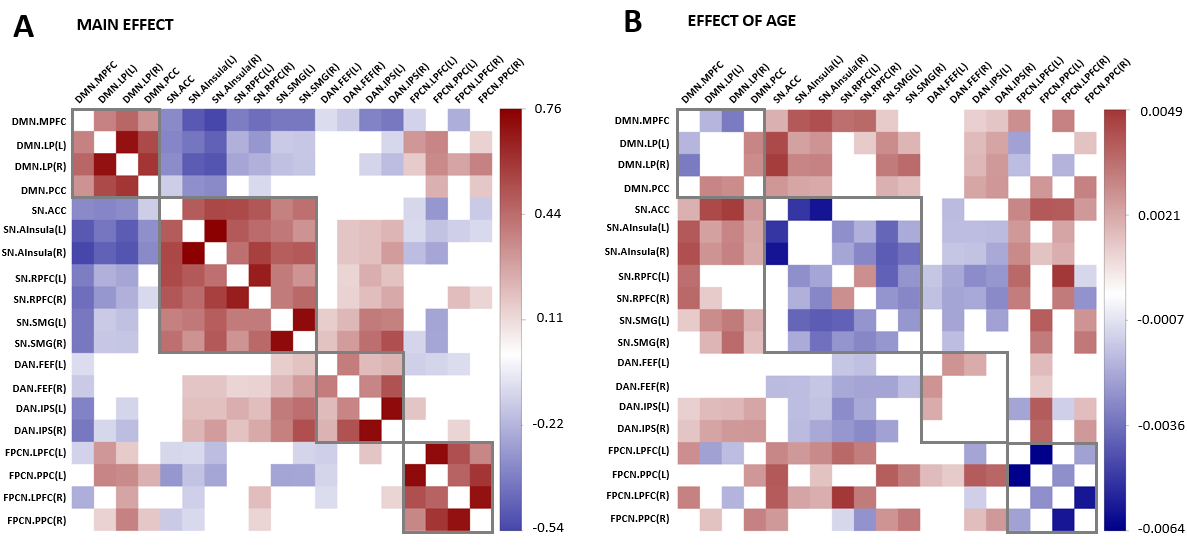


Figure S1 – Functional connectivity of cortical association networks (controlling for mean connectivity across all ROIs, head motion, WM, CSF, 4^th^ ventricle, vascular health index, and education level). Correlation matrices of functional connectivity (Pearson r, p-FDR < 0.05) for A) Main effect and B) Effect of age. Functional modules included in the analysis: default mode network (DMN), salience network (SN), dorsal attention network (DAN), and frontoparietal control network (FPCN).

Table S1 – Results of mediation analysis – Connectivity within networks

|  |  |  |  |  |  |  |  |  |
| --- | --- | --- | --- | --- | --- | --- | --- | --- |
| **MODEL 1** | | **M1 - ARAS-DMN** | | | | | | |
| **X** | **Y** |  | | | | | | |
| AGE | DMN-DMN | **Coeff** | **STE** | **t (~N)** | **Z** | **CI lb** | **CI ub** | **p** |
| **Path a1** | | -0.2 | 0.05 | -3.89 | -3.78 | -0.24 | -0.17 | 0.000 |
| **Path b1** | | 0.13 | 0.04 | 3.33 | 3.29 | 0.1 | 0.15 | 0.001 |
| **Path c'** | | 0.01 | 0.05 | 0.12 | 0.14 | -0.03 | 0.04 | 0.886 |
| **Total Effect (c)** | | -0.02 | 0.05 | -0.38 | -0.44 | -0.05 | 0.02 | 0.662 |
| **Indirect Effect (a1*b1)** | | -0.03 | 0.01 | -2.54 | -3.14 | -0.03 | -0.02 | 0.002 |
|  |  |  |  |  |  |  |  |  |
| **MODEL 2** | | **M1 - ARAS-SN** | | | | | | |
| **X** | **Y** |  | | | | | | |
| AGE | SN-SN | **Coeff** | **STE** | **t (~N)** | **Z** | **CI lb** | **CI ub** | **p** |
| **Path a1** | | -0.26 | 0.05 | -5.2 | -3.79 | -0.29 | -0.23 | 0.000 |
| **Path b1** | | 0.05 | 0.05 | 1.15 | 1.15 | 0.02 | 0.09 | 0.248 |
| **Path c'** | | -0.29 | 0.05 | -5.62 | -3.71 | -0.33 | -0.26 | 0.000 |
| **Total Effect (c)** | | -0.31 | 0.05 | -6.09 | -3.7 | -0.34 | -0.27 | 0.000 |
| **Indirect Effect (a1*b1)** | | -0.01 | 0.01 | -1.1 | -1.02 | -0.02 | -0.01 | 0.309 |
|  |  |  |  |  |  |  |  |  |
| **MODEL 3** | | **M1 - ARAS-DAN** | | | | | | |
| **X** | **Y** |  | | | | | | |
| AGE | DAN-DAN | **Coeff** | **STE** | **t (~N)** | **Z** | **CI lb** | **CI ub** | **p** |
| **Path a1** | | -0.08 | 0.05 | -1.55 | -1.52 | -0.12 | -0.05 | 0.128 |
| **Path b1** | | -0.02 | 0.04 | -0.52 | -0.47 | -0.05 | 0 | 0.637 |
| **Path c'** | | 0.12 | 0.04 | 2.78 | 2.76 | 0.09 | 0.14 | 0.006 |
| **Total Effect (c)** | | 0.12 | 0.04 | 2.83 | 2.83 | 0.09 | 0.15 | 0.005 |
| **Indirect Effect (a1*b1)** | | 0 | 0 | 0.44 | 0.88 | 0 | 0.01 | 0.378 |
|  |  |  |  |  |  |  |  |  |
| **MODEL 4** | | **M1 - ARAS-FPCN** | | | | | | |
| **X** | **Y** |  | | | | | | |
| AGE | FPCN-FPCN | **Coeff** | **STE** | **t (~N)** | **Z** | **CI lb** | **CI ub** | **p** |
| **Path a1** | | -0.06 | 0.05 | -1.27 | -1.29 | -0.1 | -0.03 | 0.196 |
| **Path b1** | | 0.07 | 0.04 | 1.52 | 1.5 | 0.04 | 0.09 | 0.133 |
| **Path c'** | | -0.29 | 0.05 | -5.82 | -3.72 | -0.32 | -0.25 | 0.000 |
| **Total Effect (c)** | | -0.29 | 0.05 | -5.9 | -3.7 | -0.32 | -0.26 | 0.000 |
| **Indirect Effect (a1*b1)** | | 0 | 0 | -0.85 | -0.51 | -0.01 | 0 | 0.608 |

Table S2 – Results of mediation analysis – Connectivity between networks

| **MODEL 5** | | **M1 - ARAS-DMN** | | | | | | |
| --- | --- | --- | --- | --- | --- | --- | --- | --- |
| **X** | **Y** | **M2 - ARAS-SN** | | | | | | |
| AGE | DMN-SN | **Coeff** | **STE** | **t (~N)** | **Z** | **CI lb** | **CI ub** | **p** |
| **Path a1** | | -0.2 | 0.05 | -3.84 | -3.76 | -0.24 | -0.17 | 0.000 |
| **Path b1** | | -0.09 | 0.03 | -2.66 | -2.76 | -0.11 | -0.07 | 0.006 |
| **Path c'** | | 0.24 | 0.04 | 5.88 | 3.63 | 0.21 | 0.27 | 0.000 |
| **Total Effect (c)** | | 0.25 | 0.04 | 6.16 | 3.67 | 0.22 | 0.28 | 0.000 |
| **Indirect Effect (a1*b1)** | | 0.02 | 0.01 | 2.09 | 2.82 | 0.01 | 0.03 | 0.005 |
| **Path a2** | | -0.26 | 0.05 | -5.23 | -3.79 | -0.29 | -0.23 | 0.000 |
| **Path b2** | | 0.04 | 0.04 | 1.01 | 1 | 0.01 | 0.07 | 0.317 |
| **Indirect Effect (a2*b2)** | | -0.01 | 0.01 | -0.97 | -0.9 | -0.02 | 0 | 0.367 |
|  |  |  |  |  |  |  |  |  |
| **MODEL 6** | | **M1 - ARAS-DMN** | | | | | | |
| **X** | **Y** | **M2 - ARAS-DAN** | | | | | | |
| AGE | DMN-DAN | **Coeff** | **STE** | **t (~N)** | **Z** | **CI lb** | **CI ub** | **p** |
| **Path a1** | | -0.2 | 0.05 | -3.87 | -3.73 | -0.24 | -0.17 | 0.000 |
| **Path b1** | | -0.07 | 0.03 | -2 | -1.96 | -0.09 | -0.04 | 0.050 |
| **Path c'** | | 0.08 | 0.04 | 2.3 | 2.27 | 0.06 | 0.11 | 0.023 |
| **Total Effect (c)** | | 0.09 | 0.04 | 2.63 | 2.53 | 0.07 | 0.12 | 0.011 |
| **Indirect Effect (a1*b1)** | | 0.01 | 0.01 | 1.75 | 2.16 | 0.01 | 0.02 | 0.031 |
| **Path a2** | | -0.08 | 0.05 | -1.57 | -1.56 | -0.12 | -0.05 | 0.118 |
| **Path b2** | | 0.05 | 0.03 | 1.47 | 1.49 | 0.03 | 0.07 | 0.137 |
| **Indirect Effect (a2*b2)** | | 0 | 0 | -0.96 | -0.75 | -0.01 | 0 | 0.455 |
|  |  |  |  |  |  |  |  |  |
| **MODEL 7** | | **M1 - ARAS-DMN** | | | | | | |
| **X** | **Y** | **M2 - ARAS-FPCN** | | | | | | |
| AGE | DMN-FPCN | **Coeff** | **STE** | **t (~N)** | **Z** | **CI lb** | **CI ub** | **p** |
| **Path a1** | | -0.21 | 0.05 | -3.89 | -3.78 | -0.24 | -0.17 | 0.000 |
| **Path b1** | | -0.04 | 0.05 | -0.79 | -0.84 | -0.07 | 0 | 0.399 |
| **Path c'** | | 0.06 | 0.05 | 1.24 | 1.2 | 0.03 | 0.09 | 0.229 |
| **Total Effect (c)** | | 0.06 | 0.05 | 1.23 | 1.18 | 0.03 | 0.09 | 0.239 |
| **Indirect Effect (a1*b1)** | | 0.01 | 0.01 | 0.75 | 0.86 | 0 | 0.02 | 0.391 |
| **Path a2** | | -0.06 | 0.05 | -1.25 | -1.27 | -0.1 | -0.03 | 0.204 |
| **Path b2** | | 0.15 | 0.05 | 3.05 | 2.92 | 0.11 | 0.18 | 0.004 |
| **Indirect Effect (a2*b2)** | | -0.01 | 0.01 | -1.12 | -1.04 | -0.02 | -0.01 | 0.296 |

| **MODEL 8** | | **M1 - ARAS-SN** | | | | | | |
| --- | --- | --- | --- | --- | --- | --- | --- | --- |
| **X** | **Y** | **M2 - ARAS-DAN** | | | | | | |
| AGE | SN-DAN | **Coeff** | **STE** | **t (~N)** | **Z** | **CI lb** | **CI ub** | **p** |
| **Path a1** | | -0.26 | 0.05 | -5.27 | -3.78 | -0.29 | -0.23 | 0.000 |
| **Path b1** | | 0.1 | 0.04 | 2.29 | 2.25 | 0.07 | 0.13 | 0.025 |
| **Path c'** | | -0.18 | 0.05 | -3.53 | -3.63 | -0.21 | -0.14 | 0.000 |
| **Total Effect (c)** | | -0.21 | 0.05 | -4.31 | -3.86 | -0.24 | -0.17 | 0.000 |
| **Indirect Effect (a1*b1)** | | -0.03 | 0.01 | -2.03 | -2.27 | -0.03 | -0.02 | 0.024 |
| **Path a2** | | -0.08 | 0.05 | -1.6 | -1.63 | -0.12 | -0.05 | 0.103 |
| **Path b2** | | 0.04 | 0.04 | 1.07 | 1.06 | 0.02 | 0.07 | 0.288 |
| **Indirect Effect (a2*b2)** | | 0 | 0 | -0.78 | -0.48 | -0.01 | 0 | 0.630 |
|  |  |  |  |  |  |  |  |  |
| **MODEL 9** | | **M1 - ARAS-SN** | | | | | | |
| **X** | **Y** | **M2 - ARAS-FPCN** | | | | | | |
| AGE | SN-FPCN | **Coeff** | **STE** | **t (~N)** | **Z** | **CI lb** | **CI ub** | **p** |
| **Path a1** | | -0.26 | 0.05 | -5.28 | -3.8 | -0.29 | -0.23 | 0.000 |
| **Path b1** | | 0.09 | 0.04 | 2.04 | 2.07 | 0.06 | 0.11 | 0.038 |
| **Path c'** | | 0.26 | 0.04 | 5.92 | 3.63 | 0.23 | 0.29 | 0.000 |
| **Total Effect (c)** | | 0.24 | 0.04 | 5.6 | 3.65 | 0.21 | 0.27 | 0.000 |
| **Indirect Effect (a1*b1)** | | -0.02 | 0.01 | -1.89 | -1.94 | -0.03 | -0.02 | 0.053 |
| **Path a2** | | -0.06 | 0.05 | -1.24 | -1.28 | -0.1 | -0.03 | 0.201 |
| **Path b2** | | -0.08 | 0.04 | -1.95 | -1.97 | -0.1 | -0.05 | 0.049 |
| **Indirect Effect (a2*b2)** | | 0 | 0.01 | 0.98 | 1.47 | 0 | 0.01 | 0.142 |
|  |  |  |  |  |  |  |  |  |
| **MODEL 10** | | **M1 - ARAS-DAN** | | | | | | |
| **X** | **Y** | **M2 - ARAS-FPCN** | | | | | | |
| AGE | DAN-FPCN | **Coeff** | **STE** | **t (~N)** | **Z** | **CI lb** | **CI ub** | **p** |
| **Path a1** | | -0.08 | 0.05 | -1.59 | -1.57 | -0.12 | -0.05 | 0.117 |
| **Path b1** | | 0.04 | 0.05 | 0.9 | 0.92 | 0.01 | 0.08 | 0.357 |
| **Path c'** | | 0.08 | 0.05 | 1.7 | 1.74 | 0.05 | 0.12 | 0.082 |
| **Total Effect (c)** | | 0.09 | 0.05 | 1.76 | 1.79 | 0.06 | 0.12 | 0.073 |
| **Indirect Effect (a1*b1)** | | 0 | 0.01 | -0.68 | -0.29 | -0.01 | 0 | 0.769 |
| **Path a2** | | -0.06 | 0.05 | -1.24 | -1.21 | -0.1 | -0.03 | 0.228 |
| **Path b2** | | -0.11 | 0.05 | -2.33 | -2.31 | -0.15 | -0.08 | 0.021 |
| **Indirect Effect (a2*b2)** | | 0.01 | 0.01 | 1.02 | 1.53 | 0 | 0.01 | 0.126 |

Table S3 – Results of mediation analysis – Connectivity within DMN (Effects of each nucleus)

| **MODEL 1** | |  | | | | | | |
| --- | --- | --- | --- | --- | --- | --- | --- | --- |
| **X** | **Y** |  | | | | | | |
| AGE | DMN-DMN | **Coeff** | **STE** | **t (~N)** | **Z** | **CI lb** | **CI ub** | **p** |
| **Path a1** | | -0.3 | 0.06 | -5.12 | -3.75 | -0.34 | -0.26 | 0.000 |
| **Path b1** | | -0.09 | 0.06 | -1.45 | -1.49 | -0.12 | -0.05 | 0.135 |
| **Path c'** | | -0.01 | 0.05 | -0.11 | -0.14 | -0.04 | 0.03 | 0.888 |
| **Total Effect (c)** | | -0.02 | 0.05 | -0.37 | -0.38 | -0.05 | 0.02 | 0.701 |
| **Indirect Effect (a1*b1)** | | 0.03 | 0.02 | 1.36 | 1.56 | 0.01 | 0.04 | 0.118 |
| **Path a2** | | -0.21 | 0.05 | -4.15 | -3.72 | -0.24 | -0.17 | 0.000 |
| **Path b2** | | 0.07 | 0.05 | 1.38 | 1.44 | 0.04 | 0.11 | 0.151 |
| **Indirect Effect (a2*b2)** | | -0.02 | 0.01 | -1.26 | -1.19 | -0.03 | -0.01 | 0.236 |
| **Path a3** | | -0.14 | 0.05 | -2.67 | -2.69 | -0.18 | -0.11 | 0.007 |
| **Path b3** | | 0.03 | 0.1 | 0.29 | 0.3 | -0.04 | 0.1 | 0.765 |
| **Indirect Effect (a3*b3)** | | 0 | 0.02 | -0.29 | -0.16 | -0.02 | 0 | 0.877 |
| **Path a4** | | -0.27 | 0.05 | -5.17 | -3.71 | -0.31 | -0.24 | 0.000 |
| **Path b4** | | 0.02 | 0.05 | 0.39 | 0.33 | -0.02 | 0.05 | 0.741 |
| **Indirect Effect (a4*b4)** | | -0.01 | 0.01 | -0.37 | -0.42 | -0.01 | 0 | 0.673 |
| **Path a5** | | -0.08 | 0.06 | -1.38 | -1.38 | -0.11 | -0.04 | 0.168 |
| **Path b5** | | 0.02 | 0.06 | 0.3 | 0.31 | -0.02 | 0.06 | 0.760 |
|  | **Indirect Effect** | 0 | 0.01 | -0.24 | -0.13 | -0.01 | 0 | 0.896 |
| **Path a6** | | -0.1 | 0.05 | -1.84 | -1.84 | -0.13 | -0.06 | 0.066 |
| **Path b6** | | 0.02 | 0.11 | 0.23 | 0.22 | -0.05 | 0.1 | 0.822 |
| **Indirect Effect (a6*b6)** | | 0 | 0.01 | -0.16 | -0.06 | -0.01 | 0 | 0.954 |
| **Path a7** | | 0.01 | 0.05 | 0.1 | 0.07 | -0.03 | 0.04 | 0.941 |
| **Path b7** | | 0 | 0.05 | -0.02 | -0.01 | -0.04 | 0.03 | 0.992 |
| **Indirect Effect (a7*b7)** | | 0 | 0 | 0.05 | 0.07 | 0 | 0 | 0.945 |
| **Path a8** | | -0.07 | 0.05 | -1.22 | -1.21 | -0.1 | -0.03 | 0.227 |
| **Path b8** | | 0.08 | 0.04 | 1.83 | 1.84 | 0.05 | 0.11 | 0.066 |
| **Indirect Effect (a8*b8)** | | -0.01 | 0.01 | -0.92 | -0.69 | -0.01 | 0 | 0.489 |
| **Path a9** | | -0.18 | 0.05 | -3.4 | -3.25 | -0.22 | -0.15 | 0.001 |
| **Path b9** | | 0.03 | 0.05 | 0.69 | 0.69 | 0 | 0.07 | 0.492 |
| **Indirect Effect (a9*b9)** | | -0.01 | 0.01 | -0.65 | -0.57 | -0.01 | 0 | 0.566 |

Mediators: Connectivity between ARAS nuclei and the Default Mode Network: M1: DR-DMN, M2: MRF-DMN, M3: MR-DMN,M4: PAG-DMN,M5: PBC-DMN,M6: PO-DMN,M7: PPN-DMN,M8: VTA-DMN,M9: LC-DMN. Nuclei of the ARAS: dorsal raphe nucleus (DR), mesencephalic reticular formation (MRF), median raphe nucleus (MR), periaqueductal gray (PAG), parabrachial complex (PBC), pontine nucleus oralis (PO), pedunculopontine tegmental nucleus (PPN), ventral tegmental area (VTA), and locus coeruleus (LC).

Table S4 – Results of mediation analysis – Connectivity between DMN and SN networks (Effects of each nucleus)

| **MODEL 5** | |  | | | | | | |
| --- | --- | --- | --- | --- | --- | --- | --- | --- |
| **X** | **Y** |  | | | | | | |
| AGE | DMN-SN | **Coeff** | **STE** | **t (~N)** | **Z** | **CI lb** | **CI ub** | **p** |
| **Path a1** | | -0.3 | 0.06 | -5.19 | -3.74 | -0.34 | -0.26 | 0.000 |
| **Path b1** | | -0.08 | 0.05 | -1.53 | -1.58 | -0.11 | -0.04 | 0.115 |
| **Path c'** | | 0.21 | 0.04 | 4.97 | 3.72 | 0.18 | 0.24 | 0.000 |
| **Total Effect (c)** | | 0.25 | 0.04 | 6.16 | 3.73 | 0.22 | 0.28 | 0.000 |
| **Indirect Effect (a1*b1)** | | 0.02 | 0.02 | 1.44 | 1.59 | 0.01 | 0.03 | 0.111 |
| **Path a2** | | -0.21 | 0.05 | -4.14 | -3.72 | -0.24 | -0.18 | 0.000 |
| **Path b2** | | 0.13 | 0.04 | 3.06 | 3.01 | 0.1 | 0.16 | 0.003 |
| **Indirect Effect (a2*b2)** | | -0.03 | 0.01 | -2.45 | -2.9 | -0.04 | -0.02 | 0.004 |
| **Path a3** | | -0.14 | 0.05 | -2.65 | -2.75 | -0.18 | -0.1 | 0.006 |
| **Path b3** | | -0.15 | 0.09 | -1.57 | -1.54 | -0.21 | -0.08 | 0.124 |
| **Indirect Effect (a3*b3)** | | 0.02 | 0.02 | 1.28 | 1.85 | 0.01 | 0.04 | 0.064 |
| **Path a4** | | -0.27 | 0.05 | -5.16 | -3.74 | -0.31 | -0.24 | 0.000 |
| **Path b4** | | -0.13 | 0.05 | -2.83 | -2.8 | -0.16 | -0.1 | 0.005 |
| **Indirect Effect (a4*b4)** | | 0.04 | 0.01 | 2.42 | 3.07 | 0.03 | 0.05 | 0.002 |
| **Path a5** | | -0.08 | 0.06 | -1.38 | -1.37 | -0.11 | -0.04 | 0.169 |
| **Path b5** | | -0.04 | 0.05 | -0.79 | -0.82 | -0.08 | 0 | 0.414 |
| **Indirect Effect (a5*b5)** | | 0 | 0.01 | 0.58 | 1.06 | 0 | 0.01 | 0.287 |
| **Path a6** | | -0.1 | 0.05 | -1.85 | -1.85 | -0.14 | -0.06 | 0.064 |
| **Path b6** | | 0.17 | 0.1 | 1.7 | 1.67 | 0.1 | 0.23 | 0.094 |
| **Indirect Effect (a6*b6)** | | -0.02 | 0.01 | -1.16 | -1.06 | -0.03 | -0.01 | 0.290 |
| **Path a7** | | 0 | 0.06 | 0.08 | 0.1 | -0.03 | 0.04 | 0.918 |
| **Path b7** | | 0.11 | 0.05 | 2.03 | 2.1 | 0.07 | 0.14 | 0.036 |
| **Indirect Effect (a7*b7)** | | 0 | 0.01 | 0.1 | 0.2 | 0 | 0 | 0.839 |
| **Path a8** | | -0.07 | 0.05 | -1.24 | -1.23 | -0.1 | -0.03 | 0.220 |
| **Path b8** | | -0.09 | 0.04 | -2.66 | -2.57 | -0.12 | -0.07 | 0.010 |
| **Indirect Effect (a8*b8)** | | 0.01 | 0.01 | 1.06 | 1.46 | 0 | 0.01 | 0.143 |
| **Path a9** | | -0.18 | 0.05 | -3.48 | -3.74 | -0.22 | -0.15 | 0.000 |
| **Path b9** | | -0.03 | 0.05 | -0.69 | -0.67 | -0.07 | 0 | 0.505 |
| **Indirect Effect (a9*b9)** | | 0.01 | 0.01 | 0.65 | 0.84 | 0 | 0.01 | 0.399 |
| **Path a10** | | -0.26 | 0.05 | -5.24 | -3.75 | -0.29 | -0.23 | 0.000 |
| **Path b10** | | 0.05 | 0.04 | 1.1 | 1.09 | 0.02 | 0.08 | 0.274 |
| **Indirect Effect (a10*b10)** | | -0.01 | 0.01 | -1.06 | -1 | -0.02 | -0.01 | 0.318 |

Mediators: Connectivity between ARAS nuclei and the Default Mode Network: M1: DR-DMN, M2: MRF-DMN,M3: MR-DMN,M4: PAG-DMN,M5: PBC-DMN,M6: PO-DMN,M7: PPN-DMN,M8: VTA-DMN,M9: LC-DMN, M10: Connectivity within Salience Network (SN). Nuclei of the ARAS: dorsal raphe nucleus (DR), mesencephalic reticular formation (MRF), median raphe nucleus (MR), periaqueductal gray (PAG), parabrachial complex (PBC), pontine nucleus oralis (PO), pedunculopontine tegmental nucleus (PPN), ventral tegmental area (VTA), and locus coeruleus (LC).

Cattell, R.B. (Raymond B., 1971. Abilities : their structure, growth, and action. Houghton Mifflin.

Hultsch, D.F., Strauss, E., Hunter, M.A., MacDonald, S.W.S., 2008. Intraindividual variability, cognition, and aging., in: The Handbook of Aging and Cognition, 3rd Ed. Psychology Press, New York,  NY,  US, pp. 491–556.

Law, E., Connelly, P.J., Randall, E., McNeill, C., Fox, H.C., Parra, M.A., Hudson, J., Whyte, L.A., Johnstone, J., Gray, S., Starr, J.M., 2013. Does the Addenbrooke’s Cognitive Examination-revised add to the Mini-Mental State Examination in established Alzheimer disease? Results from a national dementia research register. International Journal of Geriatric Psychiatry 28, 351–355. https://doi.org/10.1002/gps.3828

MacDonald, S., Hultsch, D., Dixon, R., 2008. Predicting Impending Death: Inconsistency in Speed Is a Selective and Early Marker. Psychology and aging 23, 595–607. https://doi.org/10.1037/0882-7974.23.3.595

Mioshi, E., Dawson, K., Mitchell, J., Arnold, R., Hodges, J.R., 2006. The Addenbrooke’s Cognitive Examination revised (ACE-R): A brief cognitive test battery for dementia screening. International Journal of Geriatric Psychiatry 21, 1078–1085. https://doi.org/10.1002/gps.1610

Pendlebury, S.T., Mariz, J., Bull, L., Mehta, Z., Rothwell, P.M., 2012. MoCA, ACE-R, and MMSE versus the national institute of neurological disorders and stroke-canadian stroke network vascular cognitive impairment harmonization standards neuropsychological battery after TIA and stroke. Stroke 43, 464–469. https://doi.org/10.1161/STROKEAHA.111.633586

Rittman, T., Ghosh, B.C., McColgan, P., Breen, D.P., Evans, J., Williams-Gray, C.H., Barker, R.A., Rowe, J.B., 2013. The Addenbrooke’s Cognitive Examination for the differential diagnosis and longitudinal assessment of patients with parkinsonian disorders. Journal of Neurology, Neurosurgery and Psychiatry 84, 544–551. https://doi.org/10.1136/jnnp-2012-303618

Shafto, M.A., Tyler, L.K., Dixon, M., Taylor, J.R., Rowe, J.B., Cusack, R., Calder, A.J., Marslen-Wilson, W.D., Duncan, J., Dalgleish, T., Henson, R.N., Brayne, C., Bullmore, E., Campbell, K., Cheung, T., Davis, S., Geerligs, L., Kievit, R., McCarrey, A., Price, D., Samu, D., Treder, M., Tsvetanov, K., Williams, N., Bates, L., Emery, T., Erzinçlioglu, S., Gadie, A., Gerbase, S., Georgieva, S., Hanley, C., Parkin, B., Troy, D., Allen, J., Amery, G., Amunts, L., Barcroft, A., Castle, A., Dias, C., Dowrick, J., Fair, M., Fisher, H., Goulding, A., Grewal, A., Hale, G., Hilton, A., Johnson, F., Johnston, P., Kavanagh-Williamson, T., Kwasniewska, M., McMinn, A., Norman, K., Penrose, J., Roby, F., Rowland, D., Sargeant, J., Squire, M., Stevens, B., Stoddart, A., Stone, C., Thompson, T., Yazlik, O., Barnes, D., Hillman, J., Mitchell, J., Villis, L., Matthews, F.E., 2014. The Cambridge Centre for Ageing and Neuroscience (Cam-CAN) study protocol: A cross-sectional, lifespan, multidisciplinary examination of healthy cognitive ageing. BMC Neurology 14. https://doi.org/10.1186/s12883-014-0204-1

Trueman, R.C., Brooks, S.P., Dunnett, S.B., 2012. Choice Reaction Time and Learning, in: Seel, N.M. (Ed.), Encyclopedia of the Sciences of Learning. Springer US, Boston, MA, pp. 534–537. https://doi.org/10.1007/978-1-4419-1428-6_594

Wechsler, D., 1997. Wechsler memory scale-third edition administration and scoring manual. Pearson Assessments, pp. 93–139.
